# Molecular docking based virtual screening of carbonic anhydrase IX with coumarin (a cinnamon compound) derived ligands

**DOI:** 10.6026/97320630015744

**Published:** 2019-10-31

**Authors:** Krishnamoorthy Meenakumari, Giridharan Bupesh, Shakthivel Vasanth, C.Arul Vasu, Kanniyan Pandian, Kaliyperumal Prabhu, S Prasath

**Affiliations:** 1 Research and Development Wing, Central Research Laboratory, Sree Balaji Medical College and Hospital (SBMCH), BIHER, Chrompet, Chennai-600044, India; 2 Department of Virology, King Institute of Preventive Medicine and Research, Guindy, Chennai-600032, India; 3 Department of Inorganic Chemistry, University of Madras, Guindy Campus, Tamil Nadu, India; 4 Department of Zoology, University of Madras, Guindy Campus, Tamil Nadu, India; 5 Department of Biomedical Engineering, Bharath Institute of Higher Education and Research, Chennai-605005, Tamil Nadu, India; 6 Department of Anantomy, Sree Balaji Medical College and Hospital, BIHER, Chromepet, Chennai

**Keywords:** Carbonic anhydrase IX, coumarin, GLIDE, ADME/T, induced fit docking

## Abstract

It is of interest to design carbonic anhydrase IX (CAIX) inhibitors with improved features using molecular docking based virtual high through put screening of ligands. Coumarin (a cinnamon compound with pharmacological activity) is known as a potent phytal compound blocking tumor growth. Hence, a series of 17 coumarin derivatives were designed using the CHEMSKETCH software for docking analysis with CAIX. The catalytic site analysis of CAIX for binding with ligand molecules was completed using the SCHRODINGER package (2009). Thus, 17 ligands with optimal binding features with CAIX were selected following the calculation of ADME/T properties. We report ligands #41, #42, #19 and #15 showed good docking score, glide energy and hydrogen bond interactions without vdW clash. We further show that N-(3,4,5-trimethoxy-phenylcarbamoylmethyl) designated as compound #41 have the highest binding energy (-61.58) with optimal interactions with the catalytic residues (THR 199, PRO 201, HIS 119, HIS 94) of CAIX.

## Background

Cancer is the one of the biggest disease burden in both developing and developed countries. Ligand based virtual screening and lead identification for the design of new drugs for cancer treatment is gaining momentum [1]. It is known that cancer drugs are most commonly used by many patients in the world [2]. Recently, many attempts have been made to synthesize potential anticancer medications against several known targets. CAIX is a tumor-related isozyme which is up regulated in variety of hypoxic tumor cells [3,6]. CAIX is associated with tumor development, metastasis, and gives an appropriate domain to hypoxic tumor cells survival and expansion [7,8]. Thus, carbonic anhydrases (CAs) are zinc metallo enzymes known as a cancer target [3-5].

Carbonic anhydrase IX is the only known tumor-associated carbonic anhydrase isoenzyme. It is a member of the CA family since it is generally expressed in a limited number of normal tissues, whereas it's over expression is noted on the cell surface of a large number of solid tumors, and it is linked invariably with the hypoxic phenotype and it is mediated by the transcription factor HIF-1 [9]. Moreover, CAIX associates poor responsiveness to classical radiation and chemo therapy [10]. The expression of CAIX is highly up regulated by hypoxia, and down regulated by the wild-type von Hippel-Lindau tumor suppressor protein (pVHL) [11,12]. Since CAIX has also been shown to contribute to cell proliferation, cell adhesion, pH regulation of tumor cells and malignant cell invasion [12,13], it is considered as a target for cancer diagnostics and treatment [14, 32].Design and development of inhibitors for CAIX to hypoxic tumors remains a challenge [33,34].

**Figure 1:**
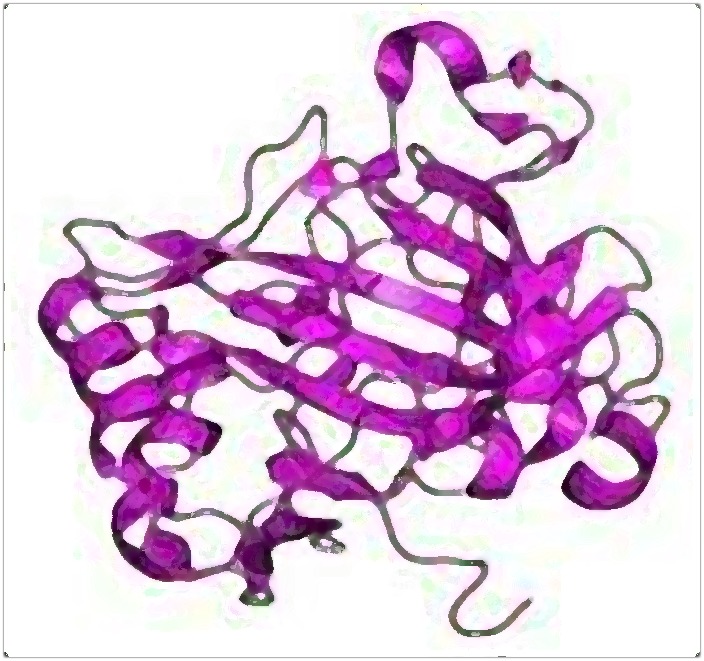
Graphical abstract for the study

The design and development of compounds as drug like compounds from natural products is classical in modern medicine [15-18]. It is of interest to design carbonic anhydrase IX (CAIX) inhibitors with improved features using molecular docking based virtual high through put screening of ligands [19,20]. Coumarin is known as a potent phytal compound from natural source blocking tumor growth. Coumarin (2H-chromen-2-one) and their derivatives are widely distributed in nature and exhibit a broad pharmacological activity [21]. Number of synthetic and natural coumarin derivatives have been reported to many studies such as antimicrobial [22,23], analgesic- anti-inflammatory (24) and anticancer activity [25-28]. A report has demonstrated that coumarin substituted benzothiazole suppresses the protein tyrosine kinase activity and the use of coumarin in cancer therapy is gaining attention [29]. Thus, we report the binding features of coumarin derivatives with CAIX in the context of cancer (Figure 1).

## Methodology

### Target

**Figure 2:**
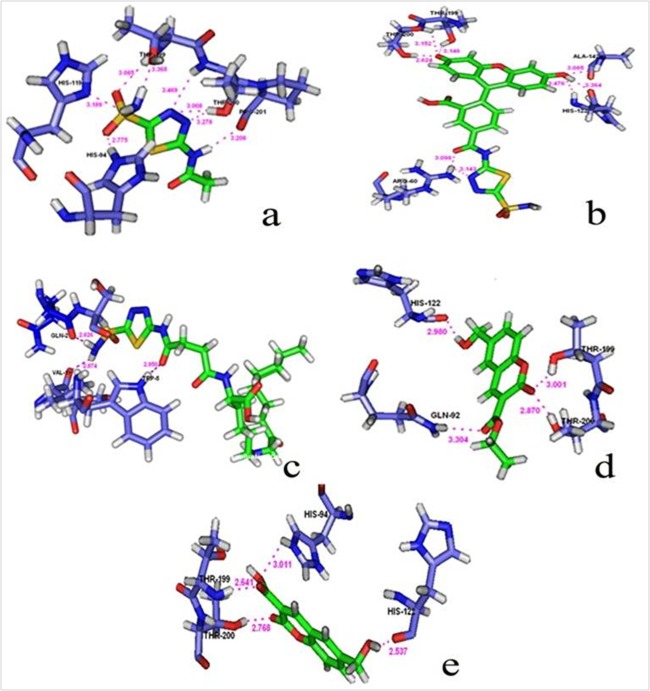
Three-dimensional crystal structure of carbonic anhydrase IX (PDB ID: 3IAI)

The protein molecule chosen for the docking studies is carbonic anhydrase-IX. The target structure data is downloaded from the Protein Data Bank (PDB; http://www.rcsb.org/ pdb/home/home.do).The crystal structure of the protein taken for the docking studies (PDB code: 3IAI) with resolution of 2.2 Å is shown in Figure 2. Water molecules are removed from the structure and hydrogen atom was added for further process.

### Ligand selection and preparation:

A total of 17 derivatives of coumarin were selected for molecular screening, based on comprehensive literature survey for natural compounds with anti-tumor activity. Ligand structures are drawn using the CHEMSKETCH software (Table 1). This is used for high throughput virtual screening of a new potential drug for CAIX. Minimization by geometric optimization using OPLS_2005 is carried out for ligands to have correct bond orders and bond angles.

### Grid generation:

Residues of each active site in CAIX were scaled with a van der Waal's radii of 1.0 Å havng partial atomic charge less than 0.25 Å. The gird was generated around active sites using QSITEFINDER and SITEMAP enclosed by a box at the center of selected residues. 

### Docking studies:

CAIX docking with coumarin was completed using the GLIDE docking tool. Glide score contains a number of parameters such as vdW, Hydrogen bond (H bond), columbic (Coul), hydrophobic (Lipo), polar interactions in the binding site (site),metal binding term (metal) and penalty for buried polar group (Burry P) and freezing rotatable bonds (RotB).

### ADME/T properties:

Absorption, Distribution, Metabolism, Excretion and Toxicity (ADME/T) properties of the docked molecules were predicted using QIKPROP tool in Schrodinger. This predicts the properties such as logBB, octanol/water partition, overall CNS activity and log IC50 values.

## Results

**Figure 3:**
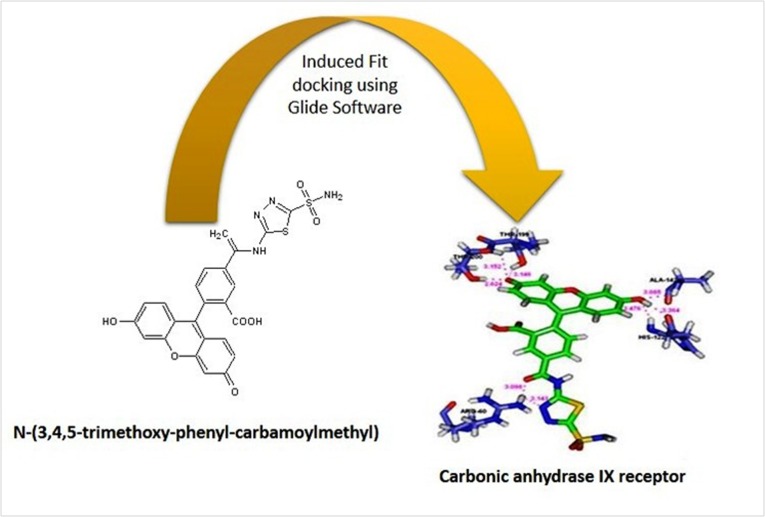
Molecular docking interaction of coumarin derivatives of (a) Native Ligand, (b) Ligand 41, (c) Ligand 42, (d) Ligand 19, (e) Ligand 15 with Target Carbonic anhydrase IX is shown.

The crystal structure of CAIX (PDB ID: 3IAI), with a resolution of 2.20 Å (Figure 2) and coumarin derivatives shown in Table 1 were used for the docking studies. The structures of ligands were drawn using the CHEMSKETCH software. Energy minimization was done by using OPLS_AA force field. The protein structure and the ligands of coumarin derivatives are subjected to High Throughput Virtual Screening (HTVS) using yje GLIDE HTVS 5 module. The possible conformations of the best ligands and native ligand along with their docking score and Glide energy is given in Table 2. HTVS 5 selected compounds were subjected to Induced Fit Docking (IFD). IFD allows the receptor to alter its binding sites to mimic the shape and binding mode of the ligand. IFD were carried out between the target protein and screened ligands using GLIDE followed by PYMOL visualization. Table 3 and Figure 3 shows the possible conformations of best ligands comparing the native ligand along with their docking score and GLIDE energy. The ADME/T properties of these compounds were further analyzed using the QIKPROP tool of Schrodinger software. This is followed by PASS prediction on the basis of activity proportional to structure.

## Discussion

Carbonic anhydrase IX has a very high catalytic activity for the hydration of carbon dioxide to bicarbonate and protons. This is used as a marker of tumor hypoxia and as a prognostic factor for many human cancers. Coumarins constitute totally a new class of inhibitors of the zinc enzyme carbonic anhydrase, which bind at the entrance of the active site. The PDB structure (PDB ID: 3IAI) and the coumarin derivatives were used for docking analysis. The ligands did not have correct bond orders and bond angles. Hence, the missing hydrogen atoms and unfilled valence atoms were corrected using the OPLS_2005 force field. The active sites are predicted using Q-SITE FINDER and SITEMAP [30,31] for further screening and docking analysis. GLIDE module from the Schrodinger package is used for docking analysis. All the ligands were minimized and subjected to High Throughput Virtual Screening (Table 2). Parameter such as G-Score, Glide Energy, H-bonds and Good Van-der-walls interactions were estimated. The more negative value of GLIDE score indicates that good binding affinity of ligand with receptor.

Five ligands with good score, energy and hydrogen bonding are selected for Induced Fit Docking studies from HTVS screening. Docking score and GLIDE energy of co-crystal ligand is (-7.54),(-43.82 kcal/mol) were calculated using IFD. Further, interactions with the residues (THR 199, PRO 201, HIS 119 and HIS 94) were observed. Ligand #41 has best score (-8.92) and energy (-61.58 kcal/mol) compared with other compounds. Hydrogen bonding with the residues (THR 200, THR 199) was seen. Ligand 42, Ligand 19 and ligand 15 also exhibit good interactions with the receptor. Ligand 42 has docking score (-8.77), GLIDE energy (-58.77 Kcal/mol) and hydrogen bonding with the residues (VAL 19, GLN 2, TRP 5). Ligand 19 has a docking score (-8.43), Glide energy (-45.12 Kcal/mol) and hydrogen bonding with the residues (GLN 92, THR 200, HIS 122, THR 199). Ligand 15 has a docking score (-7.86), Glide energy (-38.61 Kcal/mol) and interactions with the residues (THR 200, THR 199, HIS 94, HIS 122). Moreover, these compounds satisfy Lipinski's rule of five and absorption, distribution, metabolism, excretion and toxicity (ADMET) properties for further consideration. Additionally, PASS prediction shows that ligand 41 have 43 possible biological activity. It shows activity and inhibitor values for oxido reductase Pa (0,817) and Pi (0,008); diuretic Pa (0,768) and Pi (0,003) anti glaucomic Pa (0,602) and Pi (0,004) and carbonic anhydrase Pa (0,324) and Pi (0,002). It is further shown that ligand 42 shows 25 possible biological activity data (data not shown) while the native ligand shows 107 possible biological activity data (data not shown) features.

## Conclusion

We show that N-(3,4,5-trimethoxy-phenylcarbamoylmethyl) designated as compound #41 have the highest binding energy (-61.58) with optimal interactions with the catalytic residues (THR 199, PRO 201, HIS 119, HIS 94) of CAIX for further consideration and evaluation using in vitro and in vivo models.

## Figures and Tables

**Table 1:** Chemical name of the coumarin derivatives

COMPOUND	CHEMICAL NAME
Compound 5	7-methoxy-2H-chromen-2-one
Compound 6	7-ethoxy-2H-chromen-2-one
Compound 7	7-propoxy-2H-chromen-2-one
Compound 11	7-methoxy-2-oxo-2H-chromene-4-carboxylic acid
Compound 12	2-oxo-2H-chromene-3-carboxylic acid
Compound 13	6-methyl-2-oxo-2H-chromene-3-carboxylic acid
Compound 14	6-methoxy-2-oxo-2H-chromene-3-carboxylic acid
Compound 15	6-(hydroxymethyl)-2-oxo-2H-chromene-3-carboxylic acid
Compound 16	8-methoxy-2-oxo-2H-chromene-3-carboxylic acid
Compound 17	2-oxo-2H-thiochromene-3-carboxylic acid
Compound 18	methyl 6-(hydroxymethyl)-2-oxo-2H-chromene-3-carboxylate
Compound 19	ethyl 6-(hydroxymethyl)-2-oxo-2H-chromene-3-carboxylate
Compound 20	ethyl 7-methoxy-2-oxo-2H-chromene-3-carboxylate
Compound 21	6-(hydroxymethyl)- 2H-chromen-2-one
Compound 22	6-(amino methyl)- 2H-chromen-2-one
Compound 41	N-(3,4,5-trimethoxy-phenyl-carbamoylmethyl)
Compound 42	N-(2,6-dimethylphenyl-carbamoylmethyl)
Native ligand	n-(sulfamoyl-1,3,4-thiadiazol-2-yl)acetamide

**Table 2:** High throughput virtual screening results of 17 Ligands (coumarin derivatives) against the Target Carbonic anhydrase (CA IX)

Ligands	Docking Score	Glide Energy Kcal/mol	Hydrogen Bond D-H... A	Distance Å
Compound 42	-7.26	-60.53	(TRP 5) N-H...O	2.96
			(GLN 92) N-H...O	2.75
			(THR 200) O-H...O	3.4
			N-H...O (LEU 91)	3.28
Native ligand	-6.03	-36.27	(THR 199) N-H...O	2.95
			N-H...O (THR 199)	3.21
			(THR 200) O-H...O	3.33
			(GLN 92) N-H...O	3.06
Compound 21	-6.33	-28.24	O-H...O (THR 199)	2.67
Compound 15	-6.11	-29.57	(THR 199) N-H...O	2.78
			O-H...O (THR 199)	3.48
Compound 6	-5.72	-29.09	-	-
Compound 5	-5.64	-27.76	-	-
Compound 16	-5.33	-28.96	(HIS 64) N-H...O	2.81
			(HIS 94) N-H...O	2.91
Compound 22	-5.29	-25.73	N-H...O (PRO 201)	3.25
			N-H...O (THR 200)	3.16
Compound 14	-5.27	-21.06	(THR 200) N-H...O	2.88
			(THR 200) O-H...O	3.39
Compound 12	-5.1	-23.48	-	-
Compound 13	-5.05	-23.36	(THR 200) O-H...O	3.32
Compound 7	-4.91	-28.12	(THR 199) N-H...O	3.25
Compound 17	-4.9	-23.57	-	-
Compound 18	-4.86	-31.79	(HIS 64) N-H...O	3.2
			(THR 199) N-H...O	3.08
Compound 19	-4.74	-33.58	(THR 199) N-H...O	3.06
Compound 8	-3.96	-27.77	(THR 199) N-H...O	3.39
Compound 41	-3.92	-52.12	(HIS 64) N-H...O	2.76
			(GLN 92) N-H...O	2.74

**Table 3:** Induced fit docking results of 5 ligands with the target carbonic anhydrase

Ligands	Docking Score	Glide Energy Kcal/mol	Hydrogen Bond D-H...A	Distance Å
Native Ligand	-7.54	-43.82	N-H...O (THR199)	3.36
			N-H...O (THR199)	3.08
			(THR199) N-H...O	2.72
			N-H...O (PRO 201)	3.2
			(HIS 94) N-H...O	2.77
			N-H...N (HIS 119)	3.18
			(THR 200) O-H...N	3.27
			(THR 200) O-H...N	3
			(THR199) N-H...N	3.46
Ligand 41	-8.92	-61.58	(THR 199) O-H...O	3.14
			(THR 200) O-H...O	2.62
			(THR 200) N-H...O	3.15
			(ALA 142) N-H...O	3.08
			O-H...O (HIS 122)	3.36
Ligand 42	-8.77	-58.77	N-H...O (VAL 19)	2.87
			N-H...O (GLN 2)	2.62
			(TRP 5) N-H...O	2.95
Ligand 19	-8.43	-45.12	(GLN 92) N-H...O	3.3
			(THR 200) O-H...O	2.87
			O-H...O (HIS 122)	2.98
			(THR 199) O-H...O	3
Ligand 15	-7.86	-38.61	O-H...O (THR 200)	2.76
			O-H...O (THR 199)	2.64
			(HIS 94) N-H...O	3.01
			O-H...O (HIS 122)	2.53
